# Factors associated with sleep quality and duration in mothers of children with autism spectrum disorder: a cross-sectional study

**DOI:** 10.31744/einstein_journal/2026AO2004

**Published:** 2026-04-07

**Authors:** Patrícia Souza de Almeida, Carlos Eduardo Lins e Silva, Alisson Henrique de Almeida, Flavia Eloize de Souza Silva, Raphael Mendes Ritti-Dias, Ozeas Lima Lins-Filho, Breno Quintella Farah

**Affiliations:** 1 Instituto do Autismo Recife PE Brazil Instituto do Autismo, Recife, PE, Brazil.; 2 Universidade Federal Rural de Pernambuco Recife PE Brazil Graduate Program in Movement Sciences, Universidade Federal Rural de Pernambuco, Recife, PE, Brazil.; 3 Universidade Nove de Julho São Paulo SP Brazil Graduate Program in Rehabilitation Sciences, Universidade Nove de Julho, São Paulo, SP, Brazil.; 4 Universidade Federal de Pernambuco Recife PE Brazil Graduate Program in Physical Education, Universidade Federal de Pernambuco, Recife, PE, Brazil.

**Keywords:** Autistic disorder, Sleep, Sleep quality, Depression, Sedentary behavior, Mothers

## Abstract

**Objective:**

To analyze the factors associated with sleep quality and duration in mothers of children with autism spectrum disorder.

**Methods:**

A cross-sectional study was conducted with 595 mothers (37±7yrs) of children and adolescents with autism spectrum disorder (8.1±3.1yrs). The assessed sleep parameters included sleep quality and duration. Mothers reporting poor sleep quality and those who did not sleep between seven and nine hours per night were considered at risk. Demographic data (age, education, income, marital status, and number of children), child-related data, comorbidities (overweight, diabetes, hypertension, dyslipidemia, heart disease, depression symptoms, and anxiety), and lifestyle factors (physical activity, sedentary behavior, and smoking) were evaluated.

**Results:**

Forty-four percent of mothers reported poor sleep quality, and 70.4% did not achieve the recommended seven to nine hours of sleep per night. Factors associated with poor sleep quality included being unmarried [Odds-ratio(OR): 2.152; 95% confidence interval (95%CI)= 1.332-3.478], heart diseases (OR=4.859; 95%CI=1.153-20.471), depression symptoms (OR=3.833; 95%CI=(2.273-6.464), sedentary behavior on weekdays (OR=2.293; 95%CI=1.335-3.938), dissatisfaction with their child's therapy (OR=1.990; 95%CI=1.214-3.262), and inadequate sleep duration (OR=9.025; 95%CI=4.661-17.473). Inadequate sleep duration was associated with depression symptoms (OR=1.965; 95%CI=1.290-2.993), age (OR=1.051; 95%CI=1.152-2.517), number of children with autism spectrum disorder (OR=3.574; 95%CI=1.152-.517), total number of children (OR=1.655; 95%CI=1.089-2.517), and poor sleep quality (OR=6.259; 95%CI=3.843-10.193).

**Conclusion:**

Most mothers of children with autism spectrum disorder experience poor sleep quality and inadequate sleep duration. Demographic data, child-related data, heart disease, depressive symptoms, and sedentary behavior were associated with inadequate sleep.

## In Brief

Forty-four percent of mothers of children with autism spectrum disorder have poor sleep quality, and 70.4% did not meet the recommended 7-9 hours of sleep. Being unmarried, having depressive symptoms or heart disease, a sedentary lifestyle, dissatisfaction with the child's therapy, and inadequate sleep were all associated with increased odds of poor sleep quality. Age, depressive symptoms, having more children, and poor sleep were associated with sleeping less than 7 hours or more than 9 hours per night.

## Highlights

■ Forty-four percent of mothers of children with autism spectrum disorder have poor sleep quality.
■ Seventy percent did not meet the recommended 7-9 hours of sleep.
■ Demographic, child-related, and clinical data were associated with sleep problems.

## Graphical abstract

**Figure:**
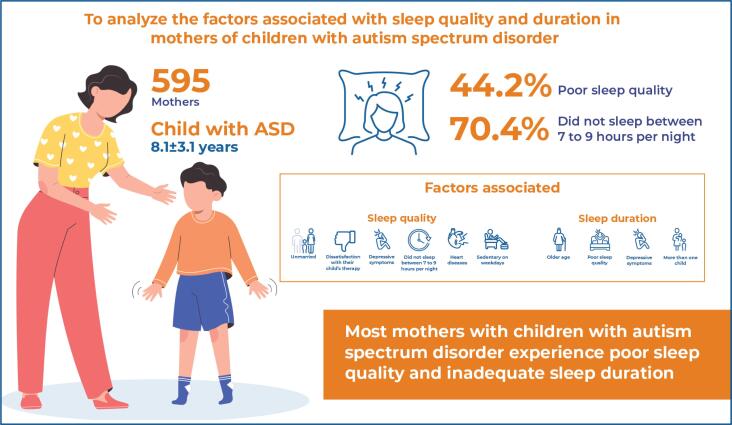

## INTRODUCTION

Children with autism spectrum disorder (ASD) are characterized by difficulties in social interaction and communication, as well as restrictive and repetitive patterns of behavior, activities, and interests.^(1)^ The diagnosis of ASD in a child affects family dynamics, increases parenting demands, and may lead to family conflict and health problems.^(2, 3)^ Mothers are generally the primary caregivers for their children, which exposes them to a greater risk of health problems such as anxiety, depression, and burnout compared with fathers.^(4–6)^

Previous studies have reported poorer sleep quality and sleep deprivation among parents of children with ASD compared to those of typically developing children.^(7,8)^ Micsinszki et al.^(9)^ found that 77.6% of parents of children with ASD reported poor sleep quality, and 70.4% of mothers did not achieve the recommended sleep duration of 7–9 hours per night.^(10,11)^

Sleep is a fundamental component of physical and mental health and is directly influenced by environmental and behavioral factors. ^(12)^ Poor sleep quality in mothers is associated with sleep disturbance in their children.^(13,14)^ However, it is still not well understood whether mothers’ personal factors (demographic characteristics, comorbid conditions, and lifestyle factors) can predict both the quality and duration of sleep in these mothers. Jenabi et al.^(13)^ found no relationship between mothers’ sleep quality and demographic data (maternal age, education, and employment) in a sample of 100 mothers, a finding also reported by Bin Eid^(14)^ in a study involving 29 mothers. Given that mothers are the most burdened in their child's care^(4–6)^ and that sleep is a determinant of health^(12)^ identifying the factors associated with sleep among mothers of children with ASD represents an important gap in the literature.

## OBJECTIVE

This study aimed to analyze factors associated with sleep quality and duration among mothers of children with autism spectrum disorder .

## METHODS

### Design and ethical issues

This cross-sectional observational study was conducted in accordance with the Strengthening the Reporting of Observational Studies in Epidemiology guidelines.^(15)^ The study was approved by the Ethics Committee of the *Universidade Federal Rural de Pernambuco* (CAAE: 82721624.1.0000.9547; #7.305.621). Written informed consent was obtained from all participating mothers.

### Setting

Mothers were recruited via social media and television programs, offering their children the opportunity to attend one-day, free-of-charge Summer Camp organized by the *Instituto do Autismo* (Pernambuco, Brazil), held from January 6 to 24, 2025. The event featured recreational activities, including sports, inflatable toys, and aquatic activities, for children and adolescents with ASD whose diagnoses were confirmed by a neurologist or pediatric neurologist.

### Participants

The inclusion criteria for this study were: a) age ≥18 years, and b) being the mother of a child or adolescent with ASD. Mothers were excluded if they refused to participate, were not biological, or were unable to answer questions related to sleep.

### Data collection

Data collection was conducted through questionnaires administered individually in an interview format in a private room, lasting approximately 15 min. The primary outcomes were sleep quality and sleep duration. Demographic data, child-related data, anxiety, depression, physical activity, and sedentary behavior were assessed as potential factors associated with sleep outcomes.

### Sleep parameters

Sleep-related problems were assessed and categorized based on sleep quality and duration. Sleep quality was evaluated using the question, "How would you rate the quality of your sleep?" The possible responses were: "poor," "fair," "good," "very good," and "excellent." Mothers who reported "poor" sleep quality were classified as having poor sleep. Regarding sleep duration, participants were asked: "In the past 30 days, how many hours of sleep did you get per night on average? (This may differ from the number of hours spent in bed)." Sleep duration was considered inadequate for mothers who reported sleeping for less than 7 hours or more than 9 hours per night.^(16)^ These sleep measurements have been previously validated against the Pittsburgh Sleep Quality Index (PSQI), with a reliability score of 0.74.^(17)^

### Comorbid conditions

The severity of depressive symptoms in mothers was assessed using the Patient Health Questionnaire-9 (PHQ-9). This instrument consists of nine items that evaluate the extent to which each situation has affected mothers over the past 14 days. Responses are rated on a Likert scale ("not at all," "several days," "more than half the days," and "nearly every day"), with total scores ranging from 0 to 27. Higher scores indicate greater severity of depressive symptoms. In the present study, mothers with scores greater than 9 were considered to have depressive symptoms.

Anxiety was assessed using the Generalized Anxiety Disorder-7 (GAD-7), which consists of seven items evaluating how each situation has affected mothers over the past 14 days. Responses are also rated on a Likert scale ("not at all," "several days," "more than half the days," and "nearly every day"), with scores ranging from 0 to 21. Higher scores reflect greater anxiety levels and mothers with scores above 9 were considered anxious. Both the PHQ-9 and GAD-7 are recommended for mental health assessments^(18,19)^ and have demonstrated good psychometric properties in the Brazilian population.^(20,21)^

Participants were also asked whether a physician had ever informed them that they had any of the following conditions: overweight, *diabetes mellitus*, hypertension, dyslipidemia, or cardiovascular disease.

### Lifestyle factors

Physical activity levels and sedentary behavior were assessed using the short version of the International Physical Activity Questionnaire (IPAQ).^(22)^ The IPAQ evaluates the amount and frequency of walking and moderate-to-vigorous physical activity over a typical week. In the present study, mothers were classified as physically active (engaging in at least 150 minutes of moderate-to-vigorous physical activity per week) or insufficiently active (less than 150 minutes per week).^(23, 24)^ Self-reported sitting time on weekdays and weekends was used as a proxy for sedentary behavior. For analysis, mothers were divided according to sitting time and categorized into quartiles, with the highest quartile considered the risk category.

Mothers were also asked whether they were current smokers or former smokers using dichotomous response options (yes/no).

### Demographic and child-related data

Information was collected on mothers’ age, education level (less than high school or high school and above), income (less than one minimum salary or one or more minimum salaries), marital status (married or unmarried), and employment status (employed or unemployed). Mothers were also asked about the number of typically developing children, number of children with ASD, the age of the child with ASD, whether the child had received therapy, their level of satisfaction with the therapy, the child's level of support needs, the availability of a support network, and the father's involvement in caregiving.

### Sample size

The minimum sample size of 552 mothers was calculated based on a population of 100,000 mothers, a prevalence of 50% (to account for the assessment of different outcomes in the project), a margin of error of 5 percentage points, a design effect (deff) of 1.2, and 95% confidence interval (95%CI). An additional 20% was added to the sample size to account for potential refusals and losses.

### Statistical analysis

All statistical analyses were performed using SPSS Statistics for Windows (IBM; v.25.0). Categorical variables were expressed as relative frequencies (%), whereas continuous variables were summarized as means with 95%CI.

Bivariate analysis using Pearson's chi-square test or independent t-test was conducted to identify potential factors associated with sleep quality and duration. Binary logistic regression analysis was used to identify factors independently associated with sleep quality and duration. Variables with a p<0.20 in bivariate analyses were tested, with only those with p<0.05 retained in the final model. The Hosmer-Lemeshow test was applied to assess model goodness-of-fit. A significance level of p<0.05 was adopted for all analyses.

## RESULTS

**Figure 1:**
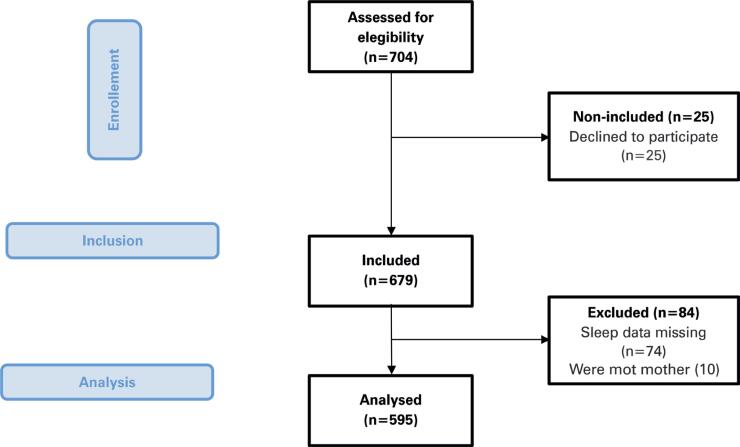
Patients selection

During the data collection period, 704 mothers were invited to participate; 25 refused to respond, 10 were not biological mothers of children and adolescents with ASD, and 74 did not answer questions related to sleep (Figure 1). The final sample for the present study consisted of 595 mothers. Table 1 presents the general characteristics of the mothers included in this study. Mothers of children with ASD reported an average sitting time of 196±179 min/d and 218±198 min/d.

**Table 1:** General characteristics of mothers included in the study

Variables	Values
Age (years)	37±7
Age of child with ASD (years)	8.1±3.1
Education (high school or more %)	83.0
Income (% less 1 minimum salary)	66.2
Marital status (married %)	38.3
Employment (%)	23.2
Depression (%)	65.7
Anxiety (%)	59.3
Overweight (%)	31.5
Hypertension (%)	18.4
Diabetes (%)	7.3
Dyslipidemia (%)	18.3
Heart diseases (%)	5.2
Smoking (%)	4.2
Insufficiently active (%)	80.2
Number of children (one %)	44.8
Children with ASD (one %)	55.2
Child's therapy (%)	66.9
Satisfaction with child's therapy (%)	69.0
Support network (%)	70.9
Support from the child's father (%)	39.3
Support level (%) (n=491)	
Level 1	35.8
Level 2	49.9
Level 3	14.3

Values are presented as mean (standard-deviation) and frequency.

ASD: autism spectrum disorder.

**Figure 2:**
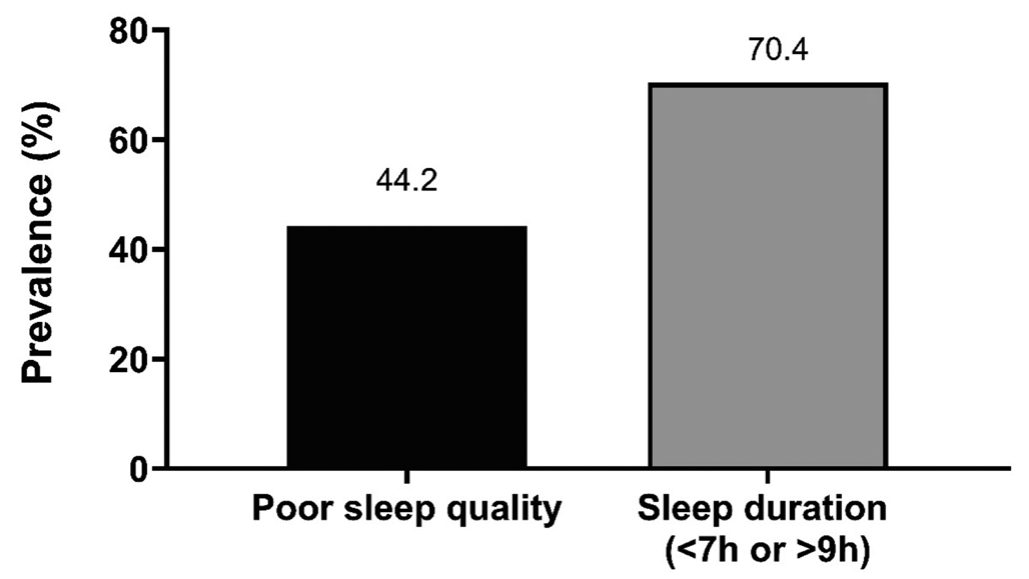
Prevalence of poor sleep quality and inadequate sleep duration in mothers of children with autism spectrum disorder

Figure 2 shows the proportion of mothers with poor sleep quality and those who reported inadequate sleep duration.

Table 2 presents the potential factors associated with sleep quality and sleep duration (p<0.20) in mothers of children with ASD. Forty-four percent of mothers reported poor sleep quality, whereas 70.4% did not sleep for 7-8 hours. The prevalence of mothers who slept less than 7 hours was 68.9%, while 3.5% of mothers slept 9 hours or more.

**Table 2:** Bivariate analyses to identify potential factors associated with sleep quality and duration in mothers of children with autism spectrum disorder

Variables	Sleep quality		p value	Sleep duration		p value
	Good	Poor		7 to 9 hours	<7h or >9	
Age (years)	36±7	37±7	0.546	35±7	37±7	0.002*
Age of child with ASD (years)	8±3	8±3	0.523	8±3	8±3	0.971
Education (high school or more %)	84.6	81.0	0.239	85.1	82.2	0.386
Income (less 1 minimum salary %)	64.8	67.9	0.416	67.4	65.8	0.699
Marital status (married %)	45.2	29.7	<0.001*	42.9	36.5	0.149*
Employment (%)	22.0	24.7	0.434	22.3	23.6	0.738
Overweight (%)	25.3	36.5	0.003*	22.3	33.4	0.007*
Hypertension (%)	15.4	19.8	0.163*	14.4	18.8	0.201
Diabetes (%)	6.6	6.1	0.788	5.7	6.7	0.646
Dyslipidemia (%)	16.6	17.9	0.687	14.9	18.0	0.364
Heart diseases (%)	3.0	7.6	0.011*	2.9	6.0	0.111*
Depression symptoms (%)	52.1	82.9	<0.001*	48.0	73.1	<0.001*
Anxiety (%)	47.3	75.3	<0.001*	44.6	66.3	<0.001*
Smoking (%)	2.4	6.5	0.014*	2.9	4.6	0.336
Insufficiently active (%)	80.9	79.0	0.563	78.9	80.6	0.662
Sedentary (%)	42.2	51.8	0.040*	25.6	23.3	0.564
Number of children (one %)	49.4	39.0	0.012*	59.2	38.4	<0.001*
Number of children with ASD (one %)	94.9	87.5	0.001*	97.7	88.9	<0.001*
Child's therapy (%)	68.1	65.4	0.491	62.9	68.5	0.183*
Satisfaction with child's therapy (%)	76.5	58.9	<0.001*	78.8	64.9	0.006*
Support network (%)	73.9	67.0	0.067*	70.7	70.9	0.951
Support from the child's father (%)	45.2	31.8	0.001*	43.7	37.3	0.148*
Support level (%) (n=491)			0.575			0.506
Level 1	37.9	33.3		32.1	37.8	
Level 2	48.5	51.6		52.9	48.4	
Level 3	13.6	15.1		15.0	13.8	

* Variables were tested using multiple binary logistic regression. Values are presented as mean (standard-deviation) and frequency.

ASD: autism spectrum disorder.

Table 3 presents the factors associated with poor sleep quality and inadequate sleep duration. Factors such as marital status (married or unmarried), presence of depressive symptoms, prolonged sedentary behavior, dissatisfaction with the child's therapy, and inadequate sleep duration were associated with a higher likelihood of poor sleep quality. Mothers with depressive symptoms, older age, a greater number of children, more children with ASD, and poor sleep quality were more likely to sleep for less than 7 hours or more than 9 hours per night.

**Table 3:** Factors associated with sleep quality and duration in mothers of children with autism spectrum disorder

Outcomes	Exposures	OR (95%CI)	Hosmer-Lemeshow test	Omnibus test
Sleep Quality (poor)	Marital status (ref: married)	2.152 (1.332-3.478)	χ^2^=2.489 p=0.928	χ^2^=136.973 p<0.001
	Heart diseases (ref: no)	4.859 (1.153-20.471)		
	Depression symptoms (ref: minimal)	3.833 (2.273-6.464)		
	Sedentary on weekdays (ref: 1st-3rd quartile)	2.293 (1.335-3.938)		
	Satisfaction with child's therapy (ref: yes)	1.990 (1.214-3.262)		
	Sleep duration (ref: 7 to 9 hours)	9.025 (4.661-17.473)		
Sleep duration (<7h or > 9h)	Depression symptoms (ref: minimal)	1.965 (1.290-2.993)	χ^2^=11.648; p=0.168	χ^2^=133.284; p<0.001
	Age (years)	1.051 (1.019-1.083)		
	Number of children with ASD (ref: one child)	3.574 (1.152-2.517)		
	Number of children (ref: one child)	1.655 (1.089-2.517)		
	Sleep quality (ref: good)	6.259 (3.843-10.193)		

OR: odds-ratio; 95%CI: 95% confidence interval; ASD: autism spectrum disorder.

## DISCUSSION

The main findings of this study were as follows: i) Nearly half of the mothers of children with ASD experienced poor sleep quality, and five out of six reported an inadequate number of hours of sleep; ii) being unmarried, presenting symptoms of depression or heart disease, reporting high sedentary behavier expressing dissatisfaction with their child's therapy, and not sleeping the recommended number of hours were all associated with a greater likelihood of poor sleep quality; and iii) depressive symptoms, older age, having more than one child (with or without ASD), and poor sleep quality were identified as factors associated with inadequate sleep duration.

In this study, 44% of mothers reported poor sleep quality. Previous studies have reported that 78% of mothers of children with ASD experience poor sleep quality.^(9,13)^ The difference observed in the results may be partly explained by the method used to assess sleep quality. In the present study, sleep quality was assessed through maternal self-perception, whereas previous studies used the PSQI, which evaluates subjective sleep quality, sleep latency, sleep duration, habitual sleep efficiency, sleep disturbances, use of sleep medication, and daytime dysfunction. Our study assessed only subjective sleep quality and could not capture all these domains. Interestingly, when we grouped the responses according to "poor" and "regular," sleep quality, the prevalence exceeded 80%, similar to the findings of other studies.^(9,13)^

The National Sleep Foundation recommends that adults sleep between 7 and 9 hours per night.^(16)^ However, in this study, more than 80% of mothers did not meet these recommendations. These findings are consistent with studies using the PSQI, which reported an average sleep duration of 6 hours per night^(9)^, as well as with studies using actigraphy,^(14)^ which showed an average sleep duration of 6 hours and 31 minutes per night.

Interestingly, we observed that sleep quality and duration were correlated; mothers who did not meet the recommended sleep duration had nearly nine times higher odds of experiencing poor sleep quality. Inadequate sleep duration results in incomplete sleep cycles, with insufficient time spent in both the REM and non-REM stages. This compromises the proper release of growth hormones,^(25)^ memory consolidation,^(26,27)^ emotional regulation,^(28)^ and activation of the glymphatic system^(29,30)^ which is responsible for the brain's metabolic waste clearance, thus impairing the perception of restorative sleep. Furthermore, both sleep quality and duration have been associated with various health factors.^(12)^ In addition, maternal health problems are widely recognized to directly affect the treatment of children with ASD, and this relationship also holds true for sleep.^(31)^

Marital status directly affected the sleep quality of these mothers, with unmarried mothers being twice as likely to report poor sleep quality compared with married mothers. These results are consistent with the findings of the longitudinal ORANJ BOWL study,^(32)^ which investigated over 2,500 individuals aged 54–72 years and found that married people had better sleep quality than those who were single, divorced, or widowed. Married mothers may receive greater social support to care for their children with ASD; however, this hypothesis requires further confirmation.

Dissatisfaction with their child's therapy was directly associated with poor sleep quality. Owing to its complexity, the treatment of children and adolescents with ASD involves intensive therapies supported by professionals from various fields, including physical education, speech therapy, occupational therapy, psychology, physiotherapy, psychometrics, and educational psychology.^(33)^ It is possible to speculate that when mothers perceive their child's therapy as incomplete (considering that 75% of the mothers in this study reported engaging in fewer than four types of therapy) it may lead to feelings of frustration, helplessness, and uncertainty about the child's future. These emotions can remain active even at night, making it difficult for mothers to achieve the level of relaxation required for adequate sleep.

Another factor associated with poor sleep quality was sedentary behavior on weekdays, with more sedentary mothers being twice as likely to experience poor sleep quality. The literature indicates that sedentary behavior is linked to sleep disorders and poor sleep quality in adults.^(34)^ To date, there has been no evidence specifically focusing on mothers of children with ASD. However, considering that sedentary behavior is often associated with increased screen time, it may lead to the suppression of melatonin secretion, disruption of the circadian rhythm, and potentially poor sleep quality.^(35,36)^

Heart disease significantly affects sleep quality, and conversely, poor sleep quality can exacerbate the progression and severity of these conditions.^(37,38)^ Insomnia and obstructive sleep apnea are common sleep disorders that negatively affect sleep quality and are frequently observed in individuals with heart disease.^(38–41)^ Moreover, symptoms such as chest pain and discomfort can disrupt normal sleep patterns, contributing to a vicious cycle in which poor sleep further compromises cardiovascular health.^(38)^

Maternal depressive symptoms are associated with shorter sleep duration and poorer sleep quality. These findings are consistent with those of other studies involving mothers of children with ASD^(42)^ and the general population.^(43)^ Although the causal relationship requires further investigation, it is plausible that the neurochemical imbalance observed in individuals with depression, characterized by reduced levels of neurotransmitters, such as dopamine and serotonin, both involved in sleep regulation, directly influences sleep quality and duration.^(44)^

Other factors associated with sleep duration were maternal age and the number of children with and without ASD. Older individuals tend to sleep for fewer hours than recommended because of physiological changes related to aging.^(45)^ In addition, caregiving overload accumulated over time in children with ASD may contribute to insufficient sleep. A greater number of children, especially those with more than one child with ASD, represent a significant increase in physical and emotional demands, making it harder to maintain healthy sleep routines. Indeed, it is well established that the main factor affecting maternal sleep is the own child's sleep.^(13,14)^ Sleep deprivation in the child, respiratory issues, and level of support needed directly impact maternal sleep quality.^(13,14)^ Therefore, the greater the number of children, the more significant the potential impact on the sleep duration.

Differences between the factors associated with sleep duration and sleep quality arise from several factors. Although related, sleep duration and quality are distinct constructs influenced by different determinants and are associated with different health outcomes. For instance, individuals may experience poor sleep quality despite obtaining sufficient sleep hours or, conversely, good sleep quality with few sleep hours. Neurobiological mechanisms such as serotonin downregulation, leading to elevated cortisol levels, may impair sleep quality without altering sleep duration^(46)^ In contrast, sleep duration has been associated with demographic variables, particularly age, as older adults generally sleep fewer hours, but do not necessarily report worse sleep quality.^(47)^ Household circumstances also contribute to reduced sleep duration. Evidence suggests that each additional child at home reduces parents’ nightly sleep by approximately 4–13 min.^(48)^

Interestingly, support level, anxiety, and insufficient physical activity were not associated with sleep quality or duration. The lack of association with support level may be explained by the fact that most participants were at Level 1 or 2, which involves supervision but not full assistance with basic needs, unlike Level 3. Insufficient physical activity showed no link, whereas sedentary behavior did, suggesting that daily movement may influence sleep. Although anxiety is often associated with poorer sleep, depressive symptoms in this sample may have played a more significant role.

This is one of the largest studies conducted on mothers of children and adolescents with ASD. However, several limitations must be acknowledged. First, the cross-sectional design prevents establishing causality between the outcomes and exposures. Sleep quality and duration were assessed using single self-report questions rather than validated instruments or direct measures (e.g., actigraphy), although the questions were similar to those used in the PSQI. Data on some comorbid conditions, such as respiratory and neurological diseases, were not collected, whereas diabetes, hypertension, overweight, dyslipidemia, and heart disease were self-reported. The sample size was calculated without a specific primary outcome, as the project encompassed multiple outcomes of interest. Finally, the absence of objective measures of physical activity and information on children's sleep quality limits the explanatory power of our findings.

## CONCLUSION

Most mothers of children with autism spectrum disorder experience sleep problems, including poor sleep quality and inadequate sleep duration. Furthermore, marital status, age, sedentary behavior, depressive symptoms, dissatisfaction with the child's therapy, and the number of children were identified as factors associated with poor sleep quality and insufficient sleep duration in these mothers.

## Data Availability

After publication, data will be available from the authors upon request – this condition is justified in the manuscript.
